# Upside down: dissecting impulsivity in attention-deficit hyperactivity disorder through genotype-phenotype association analyses

**DOI:** 10.1192/j.eurpsy.2022.590

**Published:** 2022-09-01

**Authors:** L. Balogh, A. Pulay, N. Angyal, K. Vincze, T. Kilencz, Z. Nemoda, J. Réthelyi

**Affiliations:** 1 Semmelweis University, Department Of Psychiatry And Psychotherapy, Budapest, Hungary; 2 Semmelweis University, Department Of Biochemistry And Molecular Biology, Budapest, Hungary

**Keywords:** adhd, Impulsivity, DAT1, hyperactivity

## Abstract

**Introduction:**

Better evaluation and understanding of the core symptoms have key importance both in clinical practice and the research of attention-deficit hyperactivity disorder (ADHD). One hallmark neurocognitive feature of ADHD is impaired inhibition, related to impulsivity. Given the high heritability of ADHD, the assessment of genetic background of impaired inhibition may contribute to our knowledge about the genetic background of the disorder.

**Objectives:**

In our study we investigated whether different forms of impulsivity (attentive, motor, and nonplanning) and polymorphisms in genes of the noradrenergic, serotonergic, and dopaminergic neurotransmission, i.e. dopamine transporter-1 (DAT1), cathecoloamin-O-metiltransferase (COMT), and serotonin receptor-1B (HTR1B genes show association.

**Methods:**

208 aADHD patients diagnosed according to DSM-5 criteria from a clinical sample and 142 individuals from a population sample who screened positive for aADHD were included in the study. DNA samples were genotyped for the HTR-1B gene rs1321041 and the COMT gene rs4680 SNPs, moreover the DAT-1 VNTR polymorphism. Dimensional variables for impulsivity were compared between genotypes with the Generalized Linear Model procedure corrected for sex and age, using the PLINK 1.9 statistical software.

**Results:**

The 9 repeat polymorphism in DAT1 was associated with the severity of hyperactivity, moreover, all impulsivity factors. The A allele in COMT was associated with hyperactivity and better motor inhibition activity. In carriers of the G allele in HTR1B we detected significantly higher inattention scores and increased reaction time.

**Conclusions:**

Our results support the putative role of the investigated genetic polymorphisms in the etiology of impulsivity. Nevertheless, these polymorphisms demonstrate a heterogeneous associations.

**Disclosure:**

No significant relationships.

